# Chest radiographs versus CT for the detection of rib fractures in children (DRIFT): a diagnostic accuracy observational study

**DOI:** 10.1016/S2352-4642(18)30274-8

**Published:** 2018-11

**Authors:** Susan C Shelmerdine, Dean Langan, John C Hutchinson, Melissa Hickson, Kerry Pawley, Joseph Suich, Liina Palm, Neil J Sebire, Angela Wade, Owen J Arthurs, DRIFT Study Research group, K Johnson, E McLoughlin, C Lacroix, P Sutaria, P Logan, MV Verhagen, F Arfeen, A Ljutikov, M Anjari, A Gupta, MJ Soo, G Corral Guajardo, Z Alsabban, NM Majeed, C Cuscaden, M Abdeen, YS Al-Ali, S Jerew, A Kirby, S Choi, T Gaunt, C Dodd, K Halliday, L Hartley, K Macdonald, L Preston, KA Duncan, BA Sethi, AJ Quigley, J Amarnath, JL Barber, CJ Ashwin, C Keaney, CZ Lam, E Marie, MM Perez Matta, MC Williams

**Affiliations:** a Department of Clinical Radiology, Great Ormond Street Hospital for Children, London, UK; b Department of Paediatric Pathology, Great Ormond Street Hospital for Children, London, UK; c UCL Great Ormond Street Institute of Child Health, London, UK; d Centre of Applied Statistics Courses, University College London, London, UK

## Abstract

**Background:**

Internationally, chest radiography is the standard investigation for identifying rib fractures in suspected physical abuse in infants. Several small observation studies in children have found that chest CT can provide greater accuracy than radiography for fracture detection, potentially aiding medicolegal proceedings in abuse cases; however, to our knowledge, this greater accuracy has not been comprehensively evaluated. We aimed to determine differences in rib fracture detection rates between post-mortem chest radiographs and chest CT images, using forensic autopsy as the reference standard.

**Methods:**

In this retrospective diagnostic accuracy study, we searched the Great Ormond Street Hospital (London, UK) radiology information system for all children aged 0–16 years who had a post-mortem skeletal survey (ie, full-body radiography), CT, and full autopsy between Jan 1, 2012, and Jan 1, 2017, for a purpose of death investigation. Cases were excluded if the imaging was done for a reason other than a forensic investigation or if image quality was suboptimal. Radiologists were recruited as reporters on a voluntary basis via membership databases from international radiology and post-mortem imaging societies with no specific inclusion or exclusion criteria. Reporters were sent a set of chest radiographs on a password protected and encrypted USB flash drive or via a secure filesharing website and independently reported on the presence of rib fractures, fracture location, and the confidence level of their interpretation. They were masked to the clinical information of the images. 1 month later, the same reporters were sent CTs for the same cases in a random order and asked to report on the same features. The primary objective was to compare the accuracy of detection of rib fractures by use of post-mortem chest radiographs and CTs, with autopsy data as reference standard. Accuracy was assessed by comparison of diagnostic statistics, calculated using random-intercept multilevel logistic models with reporter and patient included as cross-classified random-effects.

**Findings:**

25 cases of children (aged 1 month to 7 years), with 136 rib fractures at autopsy with paired post-mortem chest radiographs and CTs, were selected for analysis. 38 radiologists were recruited as reporters from 23 international centres; 12 (32%) were consultants, median experience of 14·5 years (range 6–27), and 26 (68%) were registrars, median experience of 4 years (range 2–9). Across all radiologists, three times as many rib fractures were correctly detected by use of chest CTs compared with chest radiography (sensitivity 44·9% [95% CI 31·7–58·9] *vs* 13·5% [8·1–21·5]; difference 31·4% [23·3–37·8; p<0·001]). Sensitivity for detection on the correct rib was higher by use of CT than by use of radiography (62·4% [95% CI 44·9–77·1] *vs* 23·1% [12·9–37·8]; difference 39·3% [31·9–42·2; p<0·001]), as was diagnosis of a patient with any rib fracture or fractures (81·5% [75·8–86·0] *vs* 64·7% [57·3–71·4]; difference 16·7% [11·5–22·2; p<0·001]). Radiologist confidence was higher when using CT images than radiographs (highest confidence rating given on 3317 [63·6%] of 5218 fractures for CT *vs* 1518 [46·6%] of 3303 on radiographs) and was a predictor for accurate fracture detection.

**Interpretation:**

Chest CT provides greater accuracy than conventional chest radiography for post-mortem rib fracture detection, irrespective of radiologist experience or fracture location, although both methods detected a substantial number of false positives. The diagnostic accuracy of CT should be studied further in live children ideally in a multicentre trial to assess the applicability of our results.

**Funding:**

Great Ormond Street Children's Charity, Medical Research Council, Royal College of Radiologists, Research Councils UK, National Institute for Health Research.

## Introduction

Infants who are thought to have sustained physical abuse often do not have directive signs or symptoms to indicate the site or extent of their injury, and medical professionals are reliant on clinical examination and imaging to discover injuries. National and international guidelines necessitate a series of radiographs of the whole body (ie, skeletal survey) to identify occult fractures and, in specific cases, an additional CT of the head.^1^

Rib fractures in infants are highly specific for physical abuse.^2^, ^3^, ^4^, ^5^ In a third of cases of abuse, rib fractures might be the only skeletal abnormality,^2^ and rib fractures are found in a third of infants who die as a result of an inflicted injury.^6^ Chest radiographs (anteroposterior and right and left oblique views) are the reference standard for detection of rib fractures as part of a skeletal survey, including follow-up radiographs 11–14 days after initial assessment.^1^ Reporting radiologists are required to accurately detect their presence, number, and location, and evidence of healing because this information often has clinical and medicolegal implications. However, rib fractures are difficult to detect on radiographs,^7^ with only moderate agreement between radiologists.^8^

**Research in context**

**Evidence before this study**

Rib fractures in infants are highly specific for physical abuse. In a third of abuse cases, rib fractures are the only skeletal abnormality, and rib fractures are found in a third of infants who die as a result of abuse. Accurate detection of rib fractures is crucial in the setting of suspected abuse. National and international guidelines require chest radiograph as the imaging standard for identification of rib fractures. We searched PubMed for publications between Jan 1, 2000, and Jan 1, 2017, using the terms “rib fractures”, “child abuse”, “radiology”, and “imaging” with no language restrictions. A few studies compared rib fracture detection rates between chest CT and radiography, and found that CT could identify substantially more injuries than radiography. However, these studies had small, unpowered sample sizes, low reporter numbers, or did not include an adequate reference standard.

**Added value of this study**

To our knowledge, no large, adequately powered study has evaluated rib fracture detection rates by use of CT in cases of suspected physical abuse on infants with use of autopsy as a reference standard. We showed that the diagnostic accuracy in rib fracture detection was better when assessed with chest CT than with chest radiography, regardless of reporter experience or the location of rib fracture. More rib fractures were correctly detected by use of CT than by use of radiography, reporters were more confident of their findings when assessing CT images than when assessing radiographs and the degree of confidence reported was a key predictor for correct fracture identification. CT had a lower specificity than radiography, with more fractures identified that were not present at autopsy; however, it is important to note that autopsy is an imperfect reference standard.

**Implications of all the available evidence**

Chest CT images have significantly higher diagnostic accuracy than chest radiographs for the post-mortem detection of rib fractures in infants. These findings should be confirmed in a larger group of live children in a multicentre trial.

One adult study suggested that chest CTs could yield higher detection rates for rib fractures than chest radiographs.^9^ CT might outperform radiography in paediatric trauma,^10^ but the dose of radiation from a CT scan is perceived to be unjustified in suspected cases of physical abuse. New CT algorithms and machine technology now allow optimisation of the dose of radiation, such that the benefit of accurate diagnostic imaging could outweigh the potential risk for the individual child.^11^ Post-mortem CT has been validated against autopsy findings for rib fractures in adults with a reported sensitivity of 58%,^12^ but preliminary studies^7^, ^13^ comparing diagnostic accuracy of CT and radiography in children have been underpowered.

The aim of this study was to compare the accuracy of post-mortem chest radiography and CT for the detection of rib fractures in children, with autopsy data as reference standard.

## Methods

### Study design and reporter recruitment

In this retrospective observational diagnostic accuracy study, we searched the radiology information system (RIS) at Great Ormond Street Hospital for Children, London, UK, for all children aged 0–16 years who had a post-mortem skeletal survey, post-mortem whole-body CT, and full autopsy for the purpose of death investigation between Jan 1, 2012, and Jan 1, 2017. Cases were excluded if imaging was done for reasons other than forensic investigation (eg, stillbirths, skeletal dysplasia) or if image quality was suboptimal. Ethical board approval was granted by the UK National Health Service (NHS) Health Research Authority, Research Ethics Committee (reference 04/Q0508/41).

All skeletal surveys at the hospital were acquired on a Ysio digital radiography imaging system with wireless detector (Siemens, Erlangen, Germany) at 3–6 mAs and at 64 kVp, and done as per Royal College of Radiologists guidelines^1^ (ie, three separate radiographs of the chest done in anteroposterior [frontal], and 45° right and left oblique views). All post-mortem CT imaging was done on a 64-slice multidetector system (Siemens), at 120 kV and at 200–350 mAs, with a pitch of 1 mm, and 0·625 mm collimation. Volumetric axial slices were 1 mm thick and all images were reconstructed with a soft tissue and bone algorithm. Only chest radiographs were extracted from the skeletal survey, and only chest CT image slices were extracted from the whole-body CT. All autopsies were done as per Royal College of Pathologists national guidelines,^14^ and by a specialist paediatric pathologist in conjunction with a forensic pathologist. At autopsy, all ribs were examined individually, and when a rib lesion or fracture was detected it was sent for rib histology as per guidelines.

Radiologists were recruited as reporters via a call for participants from the membership databases of the International Society for Forensic Radiology and Imaging (ISFRI), British Society of Paediatric Radiology (BSPR), and the post-mortem imaging taskforce of the European Society of Paediatric Radiology (ESPR). Reporters were recuited on a voluntary basis by expressions of interest sent by email. Before being enlisted as reporters, all radiologists who applied completed a data-confidentiality agreement. The radiologists' job title and years of general and paediatric reporting experience were recorded. We asked radiologists to self-define into one of two categories, either as a consultant (equivalent to faculty staff or attending staff, who have completed at least 5 years of radiology training in the UK) or as a specialist registrar (equivalent to residents or radiology trainees) who are in radiology training or doing a subspecialty fellowship. There were no specific inclusion or exclusion criteria for reporters.

### Image analysis

Two rounds of image interpretation were done by the reporters, who were masked to the clinical information. The first round was held between March 1, 2017, and April 1, 2017. A collection of sets of anonymised chest radiographs (each set containing the anteroposterior and both oblique views) were analysed by all reporters. Reporters were required to complete a form detailing the presence or absence of fractures for every individual rib in each case, and to disregard any cervical ribs that might be present on the images. For the presence of rib fractures, reporters were asked to document the fracture location (laterality, rib number, and location on the rib) and their confidence level of the presence of the fracture (a scale of 1–3: 1, not very confident; 2, moderately confident; 3, very confident). To avoid interpreting potential differences in terminology and language between reporters, the location of the rib fracture was recorded according to the reporter's perceived location around an imaginary clockface when the chest was viewed in axial section. Therefore, the 12 o'clock position was the sternum (not scorable), the 1 o'clock to 5 o'clock positions were locations along the left rib, the 6 o'clock position was the vertebral column (not scorable), and the 7 o'clock to 11 o'clock positions were locations along the right rib. The 1 o'clock and 11 o'clock positions would therefore refer to costosternal junction locations, and the 5 o'clock and 7 o'clock positions would refer to costovertebral junction locations. This method gave three categories of fracture locations: anterior fractures were at the 1, 2, 10, and 11 o'clock positions; lateral fractures were at the 3 and 9 o'clock positions; and posterior fractures were at the 4, 5, 7, and 8 o'clock positions. Reminder e-mails were sent to reporters every 2 weeks to return their analyses within the designated timeframe.

The second round of analysis commenced 1 month after completion of the first round (May 1, 2017 to June 1, 2017). The 1 month washout period was included to reduce the chance of reporters recalling radiographs from the first round. 25 chest CTs from the same patients were randomly re-ordered and sent to the reporters for assessment for the same variables. No feedback on diagnostic accuracy was distributed to any of the reporters during the study period.

All images were provided to the reporters in Digital Imaging and Communications Medicine (DICOM) format, either on a password protected and encrypted USB flash drive, or online via a password protected and encrypted filesharing website (ie, Dropbox), depending on reporter preference. Reporters were encouraged to replicate their usual reporting practice when viewing cases (ie, using dim lighting, appropriate image display equipment). Because DICOM format images were provided, reporters could change the brightness and contrast of radiographs and windowing of CT images. For all CT images, we provided axial volumetric slices, both in soft tissue and bone algorithms. Participants could reconstruct the images to best assess for rib fractures; we did not prespecify multiplanar reconstruction (MPR), maximum intensity projections (MIP), or three-dimensional volume rendering (3DVR) formats.

At imaging, a rib fracture was defined as a rib with complete cortical breach, cortical irregularity or buckling, or the presence of healing around the bone. At autopsy, a rib fracture was identified in a similar manner and, when present, excised and sent for rib histology as per national guidelines.

The following observations counted as the successful detection of a rib fracture by a reporter (ie, true positive), as confirmed by the autopsy results (reference standard): an observation recorded on the correct rib and in the correct location (anterior, lateral, or posterior); an observation recorded on the correct rib (and correct side) and not necessarily the correct location in the rib; or at least one observation recorded on a case with fractures (ie, correctly diagnosing a case with a rib fracture or fractures), even if observed in the wrong location. True positives, true negatives, false positives, and false negatives were derived from the raw data according to these three definitions.

### Outcomes

The primary objective of this study was to compare the accuracy of detection of rib fractures by use of post-mortem chest radiography and CT, with autopsy data as reference standard. The secondary objective was identification of whether reporter interpretation confidence, years of reporter experience, and location of the rib fracture resulted in different levels of accuracy between detection methods.

### Statistical analysis

We did a power calculation on the basis of our primary outcome for detection of fractures in the correct rib location, accounting for within-case correlation. We designed the study with 80% power to find a difference between sensitivity for radiography and CT with a significance level (type I error) of 5%. This calculation accounted for within-person imaging pairing^15^ and allowed intrapatient correlation of up to 20% (assuming fractures in adjacent ribs are not independent variables).

On the basis of data from Hong and colleagues,^13^ we assumed an average of 8·4 fractures per patient, pairwise detection rates of 14·9% for radiography and CT both positive, 34·9% for both negative, 14·0% for radiography positive and CT negative, 36·6% for radiography negative and CT positive, and, therefore, sensitivity estimates of 29% for radiography and 52% for CT. Using a conservative estimate of what one radiologist would report, we estimated that at least 121 fractures would need to be included in the analysis. This estimate resulted in a total of 25 cases for analysis, comprising both cases with and without rib fractures detected at autopsy.

To show the accuracy of radiography versus CT, we used diagnostic statistics (ie, sensitivity, specificity, positive predictive values, and negative predictive values) with 95% CIs derived from separate random-intercept multilevel logistic models, with reporter and patient included as complete cross-classified random-effects^16^ using the appropriate subset of the data required for that particular statistic. These logistic models have log odds of detection as the outcome, so we transformed the results to a more meaningful scale when possible (eg, sensitivity and specificity). We included the method of detection (radiography, CT) in these models as a single fixed-effect variable and provided a formal statistical comparison between detection methods for each diagnostic statistic.

We derived the sensitivity and specificity for each reporter from the random-effects of the same multilevel models. We present diagnostic summary statistics (with 95% CIs) split by location (anterior, lateral, or posterior) derived from multilevel modelling with location and modality added as fixed-effect dummy variables (plus interaction with method of detection). We fitted multilevel models, allowing for the same random-effects structure to investigate whether fixed-effect variables were associated with sensitivity and specificity. We defined the fixed-effect variables as experience level (years of experience), grade (specialist registrar or consultant), and whether these variables interact significantly with the method of detection. We used another model to investigate whether the confidence level of a reporter (rated between 1 and 3) for a specific fracture they identified was a predictor of the positive predictive value of the detection method. We did analyses using R version 3.5.1. We fitted multilevel models using the function glmer within package lme4,^17^ and we calculated confidence intervals from these models using a parametric bootstrap method provided by the function bootMer.

We did not ask the same reporters to analyse the same cases by use of the same method of detection more than once; therefore, within-reporter reliability for the same method of detection was not assessed. Given the large dataset for analysis, we anticipated that repeated measures could be detrimental to reporter recruitment and wished to preserve as many reporters as possible.

### Role of the funding source

The funder of the study had no role in the study design, data collection, data analysis, data interpretation, or writing of the report. The corresponding author and chief investigator (OJA) had full access to all the data in the study and final responsibility for the decision to submit for publication.

## Results

**Figure 1:**
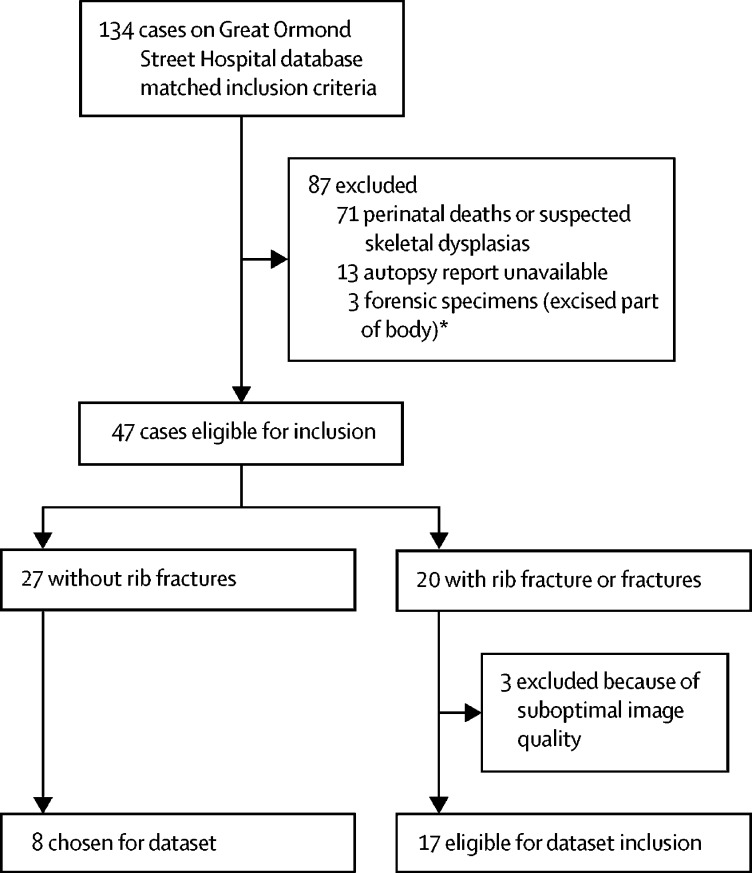
Case availability and selection *No whole-body images were available, and hence no ribs could be analysed.

Of patient records on the Great Ormond Street Hospital RIS between Jan 1, 2012, and Jan 1, 2017, we found 134 cases that had skeletal survey, post-mortem CT imaging, and autopsy. Of these cases, 71 (53%) were excluded because they were fetal deaths or stillbirth, or had suspected skeletal dysplasias; 13 (10%) did not have an authorised autopsy report; and three (2%) were images of forensic specimens (ie, an excised part of the patient's body), leaving 47 (35%) for review, of which 17 (13%) had at least one rib fracture at autopsy and matching imaging of an acceptable diagnostic quality (figure 1). We selected a further eight representative cases from the dataset of 27 patients without rib fractures to give a final sample of 25 cases. Among the 17 cases with fractures, 136 fractures were confirmed at autopsy (median number of fractures was seven, range 1–20). 111 (82%) fractures were anterior, ten (7%) were lateral, and 15 (11%) were posterior. Of the 600 ribs to evaluate (25 patients each with 24 ribs), 122 (20%) ribs had a fracture in one location and seven (1%) had multiple fractures in separate locations. Details regarding the precise number of fractures for each case and the main pathological finding or cause of death at autopsy are in table 1.

**Table 1:** Key demographic information for cases, rib fractures, and autopsy findings

	**Age**	**Sex**	**Total rib fractures**	**Likelihood of rib fractures from inflicted abuse**	**Main pathological diagnosis and autopsy comments**
1	7 years	Female	4	Unlikely	Sepsis
2	3 years	Male	20	High	Trauma, probably non-accidental injury
3	6 months	Male	6	Unlikely	Congenital vitamin D deficiency
4	13 months	Female	19	High	Trauma, probably non-accidental injury
5	1 month	Male	7	Unlikely	Small bowel mesenteric volvulus
6	1 month	Female	2	Unlikely	Sudden unexplained death in infancy, no suspicious injuries
7	2 months	Male	3	High	Trauma, probably non-accidental injury
8	4 months	Male	0	NA	Complex congenital heart disease
9	3 months	Female	8	Unlikely	Unascertained
10	11 months	Male	7	High	Asphyxia, probably non-accidental injury
11	1 month	Female	0	NA	Sudden unexplained death in infancy, no suspicious injuries
12	4 months	Female	9	Unlikely	Sudden unexplained death in infancy, no suspicious injuries
13	1 month	Male	8	Unlikely	Sudden unexplained death in infancy, no suspicious injuries
14	8 months	Female	0	NA	Acute respiratory failure, underlying syndrome
15	1 month	Male	9	Unlikely	Sudden unexplained death in infancy, no suspicious injuries
16	2 months	Female	2	Unlikely	Intracranial haemorrhage
17	1 month	Male	6	Unlikely	Sudden unexplained death in infancy, no suspicious injuries
18	1 month	Male	0	NA	Pulmonary haemorrhage
19	19 months	Female	0	NA	Unascertained
20	1 month	Male	15	High	Severe head trauma
21	8 months	Female	0	NA	Post-cardiac arrest hypoxic brain injury
22	2 months	Female	0	NA	Unascertained
23	9 months	Female	0	NA	Acquired heart disease
24	4 months	Female	4	Uncertain	Sepsis, head injury
25	4 years	Male	7	High	Trauma, probably non-accidental injury

Each of the 25 cases are summarised with autopsy findings. The likelihood of rib fracture from inflicted abuse was estimated retrospectively from the complete autopsy results. Rib fractures in children who died in non-suspicious circumstances were mainly attributed to resuscitation. NA=not applicable (for cases without rib fractures).

Of the 25 patients whose cases were included in the analysis, 12 (48%) were boys and 13 (52%) were girls, and the median age was 4 months (118 days, range 17 days to 7 years). The median time from death to imaging was 4 days (range 1–7 days), and median time from imaging to autopsy was 2 days (range 0–5 days).

38 reporters were recruited, of whom 12 (32%) were consultants with a median 14·5 years (range 6–27) of experience, and 26 (68%) were specialist registrars with a median of 4 years (range 2–9) experience. All reporters completed the first round of analysis, and 35 completed the second round of analysis. The three reporters that did not complete the second round were all consultants.

Overall sensitivity for detecting a rib fracture in the correct location was significantly higher for CT (44·9%, 95% CI 31·7–58·9) than radiography (13·5%, 8·1–21·5) with a difference of 31·4% (23·3–37·8; p<0·001; table 2). By use of CT instead of radiography, we estimate that one extra fracture would be observed for every 3·18 (95% CI 2·65–4·29) locations with fractures that are observed on autopsy (ie, number needed to treat, calculated via 100÷31·4). Specificity was slightly higher for radiography (97·9%, 95% CI 96·8–98·7) than for CT (97·0%, 95·3–98·0), with a difference of −0·9% (−1·4 to −0·6; p<0·001).

**Table 2:** Overall diagnostic performance of chest radiography and CT for rib fracture detection

	**True positive/false positive**	**False negative/true negative**	**Sensitivity (%, 95% CI)**	**Specificity (%, 95% CI)**	**Positive predictive value (%, 95% CI)**	**Negative predictive value (%, 95% CI)**
**Per fracture location**						
Chest radiography (n=68 400)	912/2391	4256/60 841	13·5% (8·1 to 21·5)	97·9% (96·8 to 98·7)	7·2% (1·9 to 23·6)	99·4% (97·0 to 99·9)
CT (n=63 000)	2089/3129	2671/55 111	44·9% (31·7 to 58·9)	97·0% (95·3 to 98·0)	12·0% (3·3 to 35·1)	99·6% (98·8 to 99·9)
Difference	..	..	31·4% (23·3 to 37·8; p<0·001)	−0·9% (−1·4 to −0·6; p<0·001)	4·8% (1·2 to 11·9 p<0·05)	0·2% (0·0 to 1·0; p≥0·05)
**Per rib**						
Chest radiography (n=22 800)	1579/1410	3323/16 488	23·1% (12·9 to 37·8)	96·4% (94·1 to 97·8)	15·9% (3·2 to 52·1)	98·1% (91·3 to 99·6)
CT (n=21 000)	2713/1886	1801/14 599	62·4% (44·9 to 77·1)	94·1% (90·5 to 96·3)	18·8% (3·9 to 56·9)	98·9% (94·8 to 99·8)
Difference	..	..	39·3% (31·9 to 42·2; p<0·001)	−2·3% (−3·7 to −1·4; p<0·001)	2·9% (0·1 to 7·4 p<0·05)	1·5% (0·1 to 3·3p<0·05)
**Per case**						
Chest radiography (n=950)	405/155	241/149	64·7% (57·3 to 71·4)	48·9% (41·6 to 56·2)	72·3% (68·5 to 75·9)	38·2% (33·5 to 43·1)
CT (n=875)	465/141	130/139	81·5% (75·8 to 86·0)	49·3% (41·8 to 56·9)	76·7% (76·7 to 73·2)	51·7% (45·7 to 57·6)
Difference	..	..	16·7% (11·5 to 22·2; p<0·001)	0·4% (−7·0 to 9·0; p≥0·05)	4·4% (−0·4 to 9·3; p≥0·05)	13·5% (6·0 to 21·4; p<0·001)

Data are shown with 95% CI and p values when available. Difference in diagnostic statistics calculated as CT minus chest radiography. The positive and negative results are the total frequencies across all reporters, cases, and locations—eg, 68 400 rib locations (anterior, lateral, and posterior) were included across all reporters on chest radiographs (three locations per rib × 24 ribs × 25 cases × 38 reporters) and 63 000 rib locations across 35 reporters on CT. 136 locations had fractures on autopsy and so a total of 136 × 38 reporters=5168 fractures could be detected on the radiographs; and 136 locations × 35 reporters=4760 fractures on CT images. Estimates are derived from multilevel models and therefore differ from the raw estimates that could be calculated from number of true positive, false positive, false negative, and true negative events.

Sensitivity for detecting a rib fracture on the correct rib and for correctly diagnosing a patient with a rib fracture or fractures was also higher by use of CT than radiography (table 2). Our results show that the sensitivity of detection on the correct rib increased by 39·3% (95% CI 31·9–42·2; p<0·001) in absolute terms from radiography to CT, and sensitivity of correctly diagnosing a patient with a rib fracture or fractures increased by 16·7% (11·5–22·2; p<0·001) in absolute terms (table 2). By use of CT instead of radiography, an estimated one additional child would be correctly diagnosed as having a rib fracture or fractures for every 6·0 (95% CI 4·5–8·7) children that would otherwise be diagnosed by use of chest radiographs (number needed to treat 100 ÷ 16·7).

**Figure 2:**
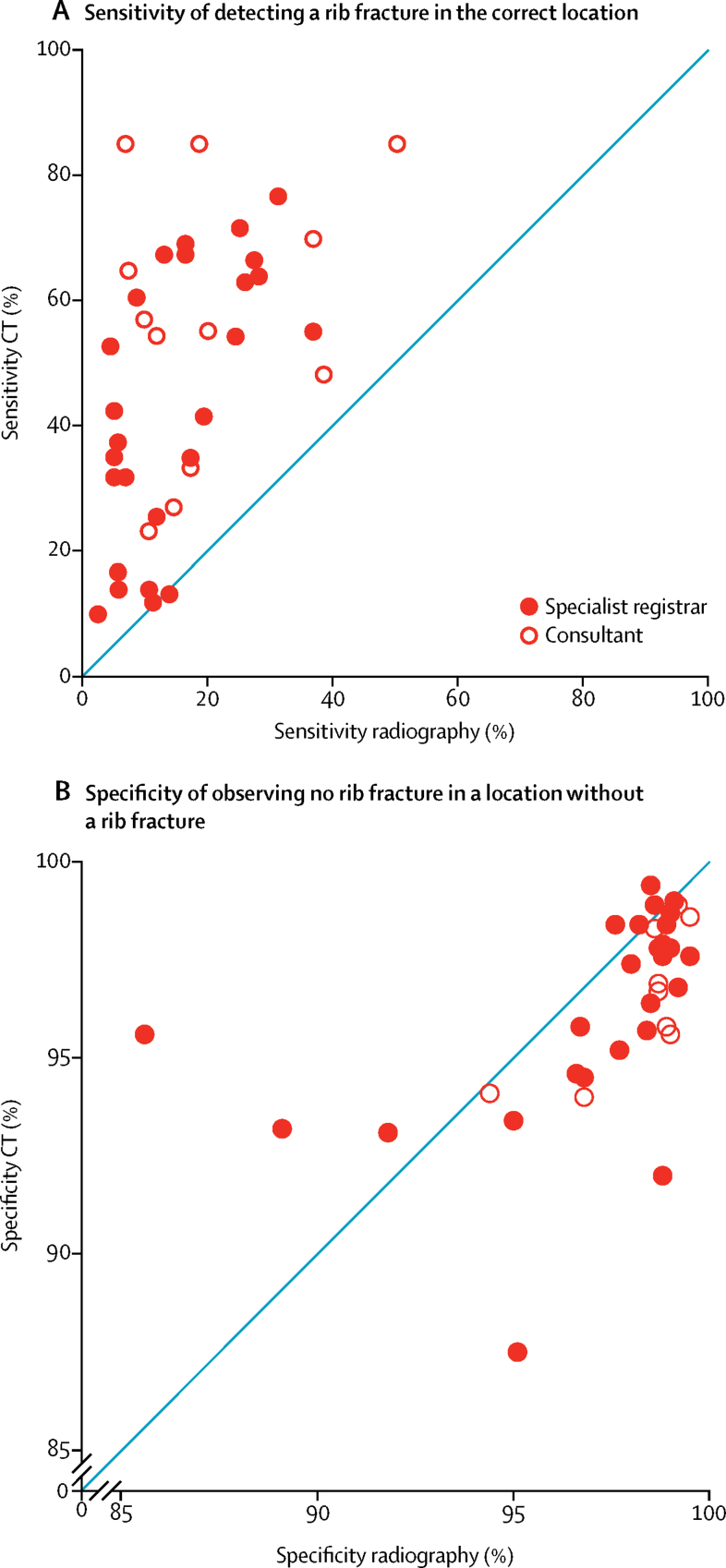
Scatter plots of sensitivity (A) and specificity (B) of CT versus chest radiography, by reporter job title Datapoints show the sensitivity and specificity of each reporter that completed both phases of the study (n=35), and those that only completed the chest radiography analysis (n=38). Estimates were derived from the random-effects of multilevel models. Graph B is on a reduced scale of 85–100%.

**Figure 3:**
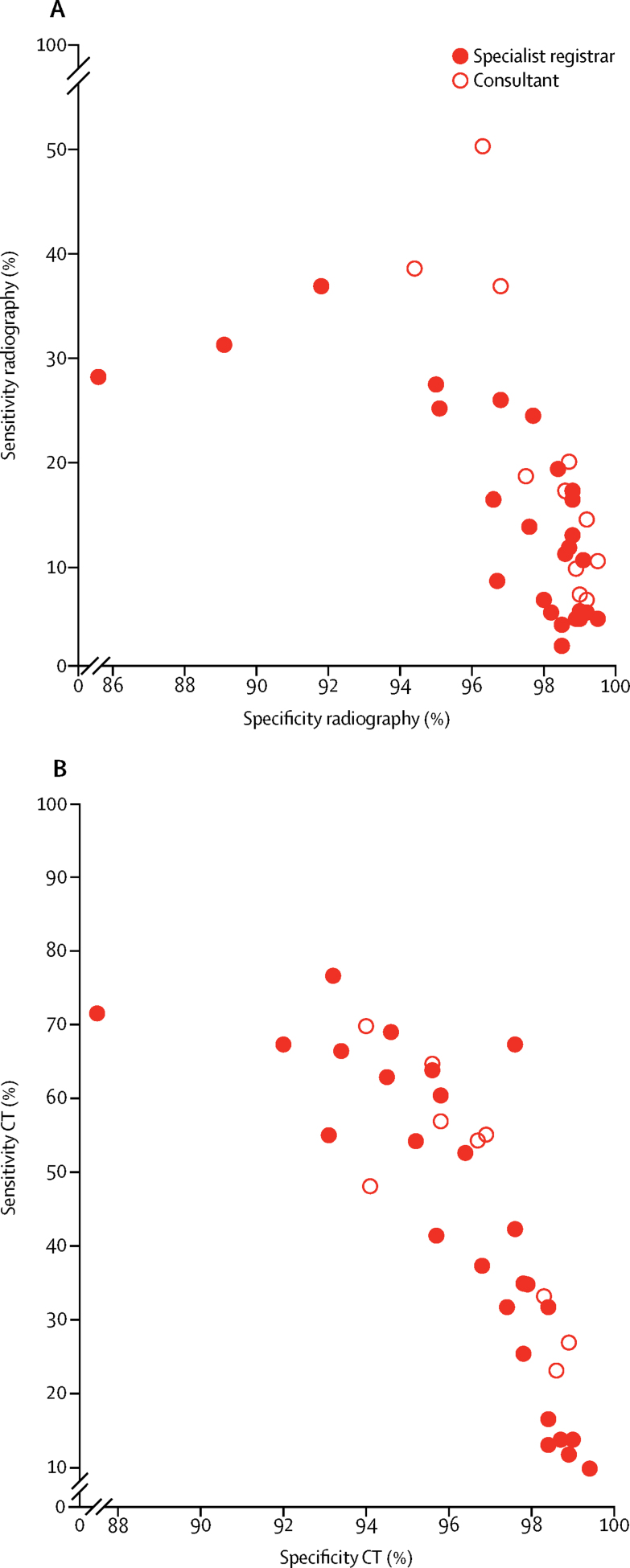
Scatter plots of sensitivity versus specificity for chest radiography (A) and CT (B) by reporter job title Datapoints show the sensitivity and specificity of each reporter that completed both phases of the study (n=35), and those that only completed the chest radiography analysis (n=38). Estimates were derived from the random-effects of multilevel models.

Sensitivity of detecting a rib fracture in the correct location was higher by use of CT than by use of radiographs for 34 (97%) of 35 reporters who completed both rounds of analysis (figure 2A). 30 (86%) of 35 reporters had slightly poorer specificity when using the CT images than when using the radiographs (on CT range 87·5–99·4 and on radiographs range 85·6–99·5; figure 2B). We saw a negative association between sensitivity against specificity for each reporter for both radiographs and CT (figure 3).

Sensitivity was highest for posterior fractures and lowest for lateral fractures for both detection methods, but significantly more fractures were found by use of CT in all fracture locations than by use of radiography (table 3; appendix). Specificity was high for all fracture locations for both detection methods.

**Table 3:** Overall diagnostic performance of chest radiography and CT by rib fracture position: anterior, posterior, and lateral

	**True positive/false positive**	**False negative/true negative**	**Sensitivity (%, 95% CI)**	**Specificity (%, 95% CI)**	**Positive predictive value (%, 95% CI)**	**Negative predictive value (%, 95% CI)**
**Anterior (n=111, 82%)**						
Chest radiography (n=22 800)	733/871	3485/17 711	15·8% (8·0 to 29·9)	97·2% (95·9 to 98·3)	14·7% (3·6 to 41·6)	98·2% (91·7 to 99·7)
CT (n=21 000)	1769/1332	2116/15 783	51·4% (32·8 to 70·0)	95·2% (92·7 to 97·0)	24·2% (6·2 to 56·6)	98·9% (94·7 to 99·8)
Difference	..	..	35·5% (24·5 to 41·7; p<0·001)	−2·0% (−3·2 to −1·3; p<0·001)	9·5% (2·5 to 16·7; p<0·01)	0·7% (0·1 to 3·0; p<0·05)
**Posterior (n=15, 11%)**						
Chest radiography (n=22 800)	88/683	482/21 547	27·3% (14·5 to 47·0)	98·4% (97·6 to 99·0)	1·6% (0·3 to 6·2)	99·9% (99·3 to 100)
CT (n=21 000)	164/1081	1011/6479	60·2% (40·3 to 78·0)	97·7% (96·5 to 98·5)	2·8% (0·6 to 10·7)	99·9% (99·4 to 100)
Difference	..	..	33·0% (23·5 to 39·4; p<0·001)	−0·7% (−1·2 to −0·4; p<0·001)	1·2% (0·1 to 4·7; p<0·05)	0 (0·0 to 0·1; p≥0·05)
**Lateral (n=10, 7%)**						
Chest radiography (n=22 800)	91/837	289/21 583	0·8% (0·0 to 2·5)	98·0% (97·0 to 98·8)	1·0% (0·2 to 4·3)	99·9% (99·6 to 100·0)
CT (n=21 000)	164/901	361/19 574	4·0% (1·1 to 10·4)	97·7% (96·5 to 98·6)	1·4% (0·3 to 5·4)	99·9% (99·7 to 100·0)
Difference	..	..	3·2% (0·6 to 8·5; p<0·05)	−0·3% (−0·6 to −0·1; p<0·01)	0·4% (−0·2 to 1·6; p≥0·05)	0 (0·0 to 0·1; p≥0·05)

Data are shown with 95% CI and p values when available. Difference in diagnostic statistics calculated as CT minus chest radiography. Diagnostic data derived according to the definition that an observation is successful if the fracture is detected on the correct rib in the correct location. The positive and negative results are the total frequencies across all reporters, cases, and locations. Estimates are derived from multilevel models and therefore differ from the raw estimates that could be calculated from number of true positive, false positive, false negative, and true negative events.

After accounting for the confidence grade and experience of each reporter by use of multilevel modelling, sensitivity remained significantly higher for CT than for radiography; the odds of detecting a rib fracture was 5·86 times greater by use of CT than by use of radiography (95% CI 4·01–8·56, p<0·001; appendix). Experience of the reporter was not a significant predictor of sensitivity and specificity for either radiography or CT (p≥0·63; appendix).

Reporters observed 3303 fractures on the chest radiographs, of which 1518 (46·6%) were observed with a confidence level of 3 (ie, very confident), and 5218 fractures were observed on the CT images, of which 3317 (63·6%) were observed with a confidence level of 3. Confidence levels were significantly higher on average for CT images than radiographs (p<0·001; appendix). Level of confidence was a significant predictor of the positive predictive value of both radiographs and CT images, with the likelihood of a fracture being correctly identified being 1·67 times greater by use of CT (95% CI 1·36–2·04; p<0·001) and 3·18 times greater on radiography (2·44–4·16; p<0·001) if the reporter has a confidence level of 3 (derived by refitting the model with CT as the reference, appendix).

Of the 35 reporters who took part in both stages of the study, the same fracture was identified by use of both CT and radiography 1280 times (irrespective of autopsy findings; appendix). Of these fractures seen by both detection methods, 531 (41·5%) were also present at autopsy. 3938 fractures were observed with radiography and that were not with CT (of which 1558 [39·6%] were present at autopsy), and 1742 fractures were only seen by use of CT and not by use of radiography (of which 275 [15·8%] were present at autopsy).

## Discussion

In this study, we have shown that chest CT outperforms conventional chest radiography in almost all aspects for the post-mortem detection of rib fractures, using autopsy as a reference standard. We saw significantly improved sensitivity for all reporters when using CT compared with when using radiography, with a slight decrease in specificity, which was mainly due to more rib fractures (both true and false) being reported by use of chest CT images than radiographs. Rib fractures in all locations were more likely to be detected by use of CT than by use of radiography, and we saw no effect of reporter experience or confidence grade on the likelihood of detection by one method over the other. Overall, our data indicate that chest CT would provide greater accuracy than radiography in the post-mortem investigation of rib fractures. The diagnostic accuracy of CT should be studied in live children to assess the wider applicability of our results.

The higher sensitivity, lower specificity, higher positive predictive value, and lower negative predictive value for rib fracture detection by use of chest CT than chest radiography seen in this study is similar to what has been found in adult studies with a similar design. Schulze and colleagues^12^ reported an overall sensitivity of 63% and specificity of 97% for post-mortem CT examinations in rib fracture detection, and Chapman and colleagues^9^ found that chest radiographs missed 75% of rib fractures seen on chest CT and that CT images detected three times as many fractures as chest radiographs. In our study we found a low sensitivity of radiography, at 27·3% or less. Reasons for this low sensitivity could include increased pulmonary atelectasis and overlying bronchopulmonary vascular markings, which could obscure subtle findings in radiographs. However, we anticipated potential reporting bias since the reporters were only asked to report on the detection of rib fractures in post-mortem cases (which might be expected to maximise both true and false positive results). Given the importance of finding rib fractures in child abuse investigations, the diagnostic accuracy of our reporters does not support continued use of radiography alone, especially in situations in which radiography findings are negative or diagnostic uncertainty exists.

The binary choice between whether the patient has a rib fracture or not is crucial in determining the further evaluation of a child as part of a child abuse investigation, and our results imply that detecting a rib fracture or fractures in one extra child for every six children imaged is too high to rely on radiography alone. Our results are in keeping with other studies with different limitations. The limitations of chest radiography for the investigation of rib fractures are recognised by experienced reporters,^18^ and CT has been proposed as an improved method of rib fracture detection.^19^ Three previous studies comparing CT with radiography for rib fracture detection have either been case series^20^ in live children without autopsy correlation,^7^ or underpowered with single-reporter bias.^13^ Wootton-Gorges and colleagues^7^ suggested that CT was superior for all rib fracture locations (anterior, lateral, and posterior), because only 79 (60%) of 131 fractures identified by use of CT were found by use of radiography in 12 live children, although only four children had complete CT scans of the chest and none had autopsy correlation. Hong and colleagues^13^ tested two radiologists with different levels of experience on an opportunistic series of 13 post-mortem radiographs and CT scans of children in a non-powered study. They found 101 confirmed fractures at autopsy in 12 patients, but the more senior radiologist only assessed the patients with rib fractures and they had few false positives. The more senior radiologist detected more rib fractures overall, and more by use of CT than radiography (85% *vs* 46%) than the first radiologist (51% *vs* 29%) although they both had wide confidence intervals. Hong and colleagues^13^ suggested in their study an effect of seniority, which could have led to the reluctance of the senior radiologist to use CT in this instance because a CT scan might be unnecessary if the diagnostic accuracy of an experienced individual is high. Our blinded study shows that CT is of benefit regardless of radiologist experience—ie, improved accuracy was seen by use of CT for reporters of all levels of experience.

Although we did not assess radiation dose, efforts have been made to establish low dose CT protocols for detection of rib fractures in live children. A case series^20^ of four children described the use of CT in suspected child abuse investigations in which the chest radiographs were entirely negative, and suggested a CT protocol with an estimated dose only two to three times the effective dose of a four-view chest radiograph (frontal, lateral, and two oblique views). Although oblique views of the chest can improve the number of fractures detected,^21^ CT provides the additional opportunity for three-dimensional reconstruction and visualisation, which can be used to show positive findings to clinicians, parents, judges, or jurors.^19^ The addition of multiplanar reconstruction with curved reformats further improves fracture detection in adults (10% improvement in sensitivity, from 71·5% to 80·9%^22^) and thus additional improvements in CT fracture detection could be possible through more advanced visualisation.

The standard radiography survey of the infant skeleton must include follow-up radiographs,^1^ because acute rib fractures can be difficult to detect and callus formation within 11–14 days of injury makes healing rib fractures detectable on chest radiographs. Follow-up radiographs detect new fractures in approximately 8–28% of cases that return.^23^, ^24^ This follow-up period presents a temporal medicolegal challenge for the police and social services, because the child needs to be in a safe place and potential perpetrators need to be identified quickly. Together with evidence that hospitals, social workers, parents, and children adhere poorly to the schedule of follow-up radiographs,^24^, ^25^ we argue that the use of CT in the acute setting in which radiographs are negative or unequivocal will probably identify otherwise undetected rib fractures, providing immediate diagnostic information, which is preferable to waiting for follow-up radiographs. Because all infants suspected of physical abuse will undergo a head CT to look for intracranial injury as per national guidelines,^1^ we argue that the addition of a subsievert chest CT during the same attendance would be pragmatic.

The location of a rib fracture is widely believed to be of diagnostic and medicolegal importance. Fractures of the anterior ribs are sometimes seen as sequelae of cardiopulmonary resuscitation, with an overall occurrence in less than 8% of cases by use of radiography.^26^, ^27^, ^28^ Our study shows anterior rib fractures are more common than previously reported on both imaging methods.

Posterior rib fractures are more widely accepted to have a higher specificity for abuse,^4^, ^6^, ^29^ and have an important role in child abuse investigations. Our finding that CT improved the proportion of posterior rib fractures detected when compared with radiography (60·2% *vs* 27·3%), further supports the use of CT for rib fracture detection in this setting.

Our study has several limitations. First, our dataset is based on post-mortem imaging and not live children, although we did intentionally choose this dataset to provide autopsy as a definitive reference standard. We do not believe the bony appearances or quality of radiographs vary substantially between live patients and post-mortem cases; however, our patient population and imaging parameters (higher dose than would be used with a live patient, no patient movement) could have contributed to our results. Further work in live children comparing chest radiographs and CT images is needed (with follow-up skeletal radiographs showing healing fractures, or autopsy in children who subsequently die, as possible reference standards). This work would help to identify whether improved accuracy for the detection of rib fractures holds true, particularly when the prevalence of rib fractures might be different (eg, potentially fewer resuscitation-induced fractures and less severe traumatic injuries that did not result in death, unlike for post-mortem cases).

Second, although we used forensic autopsy as our reference standard for the presence of rib fractures, this method could be a flawed standard. Examination of the entirety of each rib at autopsy is a challenge without dismembering the whole ribcage, which might not have occurred in every case in our series. The high number of false positives in our study (ie, detected by use of radiography or CT and not at autopsy) could have been real fractures missed at autopsy. However, we used this reference standard rather than comparing radiographs to CT images when no reference standard would be available. Another reason for high numbers of false-positive results could have included calling areas of subtle irregularity at the anterior rib ends on CT fractures.

Third, potential learning bias could have occurred, because the radiography dataset was analysed before the CT dataset for all reporters. In our study design, we considered randomising the order of presentation; however, that any investigation of non-accidental injury would involve a CT without an accompanying radiograph is not plausible. Although reviewing one imaging method in isolation (ie, CT without radiography) is not reflective of real-life practice, we wanted to know the accuracy of one imaging method compared with the other and thus asked reporters to score these separately. Potentially, combined analysis of the two detection methods could provide even more accurate results than each alone.

Fourth, reporters with more experience might have spent less time analysing the studies because of busier schedules than the reporters with less experience, thereby making their analyses less accurate, which could explain the absence of experience effect. Senior radiologists should have more experience reporting on chest radiographs, yet the proportion of true positive detections remained low. We asked all reporters to replicate their own working environment and they were able to alter image parameters and reconstructions; however, we were not able to investigate how the reporters did their readings. Nonetheless, the magnitude of differences in our results between the two detection methods still suggests that CT is superior to chest radiography for rib fracture detection.

This study shows that chest CT images give higher diagnostic accuracy than chest radiographs for the detection of rib fractures, irrespective of reporter experience or fracture location. We recommend the use of chest CT in the post-mortem evaluation of suspected cases of physical abuse of children, and chest CTs could be considered in live children when chest radiography is negative or challenging. A study into the diagnostic accuracy of chest CT in live children is indicated.

## References

[1] Royal College of Radiologists, The Society and College of Radiogaphers (2017). The radiological investigation of suspected physical abuse in children. https://www.rcr.ac.uk/system/files/publication/field_publication_files/bfcr174_suspected_physical_abuse.pdf.

[2] Barsness KA, Cha ES, Bensard DD (2003). The positive predictive value of rib fractures as an indicator of nonaccidental trauma in children. J Trauma.

[3] Kemp AM, Dunstan F, Harrison S (2008). Patterns of skeletal fractures in child abuse: systematic review. BMJ.

[4] Bulloch B, Schubert CJ, Brophy PD, Johnson N, Reed MH, Shapiro RA (2000). Cause and clinical characteristics of rib fractures in infants. Pediatrics.

[5] Cadzow SP, Armstrong KL (2000). Rib fractures in infants: red alert! The clinical features, investigations and child protection outcomes. J Paediatr Child Health.

[6] Kleinman PK, Marks SC, Nimkin K, Rayder SM, Kessler SC (1996). Rib fractures in 31 abused infants: postmortem radiologic-histopathologic study. Radiology.

[7] Wootton-Gorges SL, Stein-Wexler R, Walton JW, Rosas AJ, Coulter KP, Rogers KK (2008). Comparison of computed tomography and chest radiography in the detection of rib fractures in abused infants. Child Abuse Negl.

[8] Buesser KE, Leventhal JM, Gaither JR (2017). Inter-rater reliability of physical abuse determinations in young children with fractures. Child Abuse Negl.

[9] Chapman BC, Overbey DM, Tesfalidet F (2016). Clinical utility of chest computed tomography in patients with rib fractures CT chest and rib fractures. Arch Trauma Res.

[10] Renton J, Kincaid S, Ehrlich PF (2003). Should helical CT scanning of the thoracic cavity replace the conventional chest x-ray as a primary assessment tool in pediatric trauma? An efficacy and cost analysis. J Pediatr Surg.

[11] Lee C, Pearce MS, Salotti JA (2016). Reduction in radiation doses from paediatric CT scans in Great Britain. Br J Radiol.

[12] Schulze C, Hoppe H, Schweitzer W, Schwendener N, Grabherr S, Jackowski C (2013). Rib fractures at postmortem computed tomography (PMCT) validated against the autopsy. Forensic Sci Int.

[13] Hong TS, Reyes JA, Moineddin R, Chiasson DA, Berdon WE, Babyn PS (2011). Value of postmortem thoracic CT over radiography in imaging of pediatric rib fractures. Pediatr Radiol.

[14] Jayaram S (Nov 30, 2016). New guidelines for the investigation of sudden unexplained death in infancy. https://www.rcpath.org/discover-pathology/news/new-guidelines-for-the-investigation-of-sudden-unexpected-death-in-infancy-launched.html.

[15] Gonen M (2004). Sample size and power for McNemar's test with clustered data. Stat Med.

[16] Luo W, Kwok OM (2009). The impacts of ignoring a crossed factor in analyzing cross-classified data. Multivariate Behav Res.

[17] Bates D, Mächler M, Bolker B, Walker S (2015). Fitting linear mixed-effects models using lme4. J Stat Software.

[18] Kleinman PK, Kleinman PK (1998). Skeletal imaging strategies. Diagnostic imaging of child abuse.

[19] Berdon WE, Feldman KW (2012). A modest proposal: thoracic CT for rib fracture diagnosis in child abuse. Child Abuse Negl.

[20] Sanchez TR, Lee JS, Coulter KP, Seibert JA, Stein-Wexler R (2015). CT of the chest in suspected child abuse using submillisievert radiation dose. Pediatr Radiol.

[21] Hansen KK, Prince JS, Nixon GW (2008). Oblique chest views as a routine part of skeletal surveys performed for possible physical abuse—is this practice worthwhile?. Child Abuse Negl.

[22] Ringl H, Lazar M, Töpker M (2015). The ribs unfolded – a CT visualization algorithm for fast detection of rib fractures: effect on sensitivity and specificity in trauma patients. Eur Radiol.

[23] Harper NS, Lewis T, Eddleman S, Lindberg DM (2016). Follow-up skeletal survey use by child abuse pediatricians. Child Abuse Negl.

[24] Anilkumar A, Fender LJ, Broderick NJ, Somers JM, Halliday KE (2006). The role of the follow-up chest radiograph in suspected non-accidental injury. Pediatr Radiol.

[25] Patel H, Swinson S, Johnson K (2017). Improving national standards of child protection skeletal surveys: the value of College guidance. Clin Radiol.

[26] Bush CM, Jones JS, Cohle SD, Johnson H (1996). Pediatric injuries from cardiopulmonary resuscitation. Ann Emerg Med.

[27] Franke I, Pingen A, Schiffmann H (2014). Cardiopulmonary resuscitation (CPR)-related posterior rib fractures in neonates and infants following recommended changes in CPR techniques. Child Abuse Negl.

[28] Maguire S, Mann M, John N (2006). Does cardiopulmonary resuscitation cause rib fractures in children? A systematic review. Child Abuse Negl.

[29] Kleinman PK, Schlesinger AE (1997). Mechanical factors associated with posterior rib fractures: laboratory and case studies. Pediatr Radiol.

