# Supplementary material for: Chest radiographs versus CT for the detection of rib fractures in children (DRIFT): a diagnostic accuracy observational study
Source: Lancet Child Adolesc Health. 2018 Nov;2(11):802–11. doi: 10.1016/S2352-4642(18)30274-8 (PMC6350458; doi:10.1016/S2352-4642(18)30274-8)

THE LANCET
Child & Adolescent Health

Supplementary appendix

This appendix formed part of the original submission and has been peer reviewed. We post it as supplied by the authors.

Supplement to: Shelmerdine SC, Langan D, Hutchinson JC, et al. Chest radiographs versus CT for the detection of rib fractures in children (DRIFT): a diagnostic accuracy observational study. *Lancet Child Adolesc Health* 2018; published online Sept 21. http://dx.doi.org/10.1016/S2352-4642(18)30274-8.

Web Extra Material

Table S1: Multilevel logistic regression results of sensitivity and specificity with predictor variables

Multilevel models include patient and reporter as complete cross-classified random-effects. Sensitivity model is based on all 136 rib locations with a fracture across 25 cases, 38 reporters on radiograph, and 35 on CT (i.e. 136 x (35+38) = 9,928 rows of data). Specificity model based on the remaining 1664 rib locations without fracture (i.e. 1664 x (35+38) = 121,472 rows of data). The above predictors were added as fixed effects, including interactions between experience/grade and modality. OR= odds ratio.

	**Sensitivity**			**Specificity**		
	**Estimate**	**OR [95% CI]**	**p-value**	**Estimate**	**OR [95% CI]**	**p-value**
**(Intercept)**	-2·63	-	-	3·72	-	-
**Modality: CT (ref=Chest radiography)**	1·77	5·86 [4·01, 8·56]	<0·001	-0·33	0·73 [0·65, 0·81]	<0·001
**Grade: Cons (ref=Registrar)**	0·68	1·97 [0·76, 5·11]	0·16	0·29	1·33 [0·60, 2·96]	0·47
**Experience level (years)**	-0·02	0·98 [0·92, 1·05]	0·63	0·01	1·01 [0·95, 1·07]	0·79
**Modality*experience**	-0·02	0·98 [0·96, 1·01]	0·203	0·01	1·00 [0·99, 1·02]	0·73
**Modality*grade**	0·03	1·03 [0·69, 1·54]	0·89	-0·46	0·63 [0·49, 0·80]	<0·001

Table S2: Positive predictive value of successful rib fracture detection with predictor variables from multilevel logistic regression results.
Diagnostic data derived according to definition 1 – i.e. where an observation is 'successful' if the fracture is detected on the correct rib and in the correct location. Models based on all rib fracture observations made by all reporters across both modalities (i.e. 3303 observations on chest radiograph + 5218 observations on CT = 8521 rows of data).Multilevel models include patient and reporter as complete cross-classified random-effects. Modality confidence level, experience and grade added as fixed effects (with interaction).

	**Estimate**	**OR [95% CI]**	**p-value**
**(Intercept)**	-4·68	-	-
**Modality: CT (ref=Chest radiography)**	1·90	6·70 [4·12, 10·89]	<0·001
**Confidence level 2 (ref=1)**	0·73	2·08 [1·57, 2·76]	<0·001
**Confidence level 3 (ref=1)**	1·16	3·18 [2·44, 4·16]	<0·001
**Grade: Cons (ref=Registrar)**	0·99	2·69 [1·72, 4·20]	<0·001
**Experience level (years)**	0·01	1·01 [0·98, 1·04]	0·507
**Modality*experience**	-0·03	0·97 [0·94, 1·00]	0·069
**Modality*grade**	-0·53	0·59 [0·38, 0·91]	0·016
**Modality*confidence level 2**	-0·47	0·63 [0·44, 0·90]	0·012
**Modality*confidence level 3**	-0·65	0·52 [0·38, 0·72]	<0·001

Table S3: Proportion of true positives for rib fractures detected at chest radiography and/or CT across all readers compared to those reported at autopsy. The denominator represents those fractures identified at either chest radiography and/or CT imaging, the numerator represents the number of fractures present at autopsy. The numbers represent of the total fractures across all 35 readers in this study.

		CT	
		**Fracture observed**	**Fracture not observed**
**Chest Radiography**	**Fracture observed**	531 / 1280 (41.5%)	1558 / 3938 (39.6%)
	**Fracture not observed**	275 / 1742 (15.8%)	2396 / 56040 (4.3%)

Figure S1:
Example of false positive rib fracture diagnosis on chest radiography, true negative on CT (Case Code 11). Post-mortem imaging of a 17-day-old female patient, obtained 3 days after death. (a) Anteroposterior and (b) left oblique chest radiographs demonstrate an irregularity of the left third rib anteriorly (arrow) which was reported as a healing fracture by 31/38 (81.5%) readers. The corresponding volume rendered post-mortem chest CT of the chest (c) reveals the lesion to be an irregularity of rib contour (dashed arrow) rather than a fracture. Incidental note is also made of a left 7th anterior bifid rib, confirmed at autopsy (not shown).

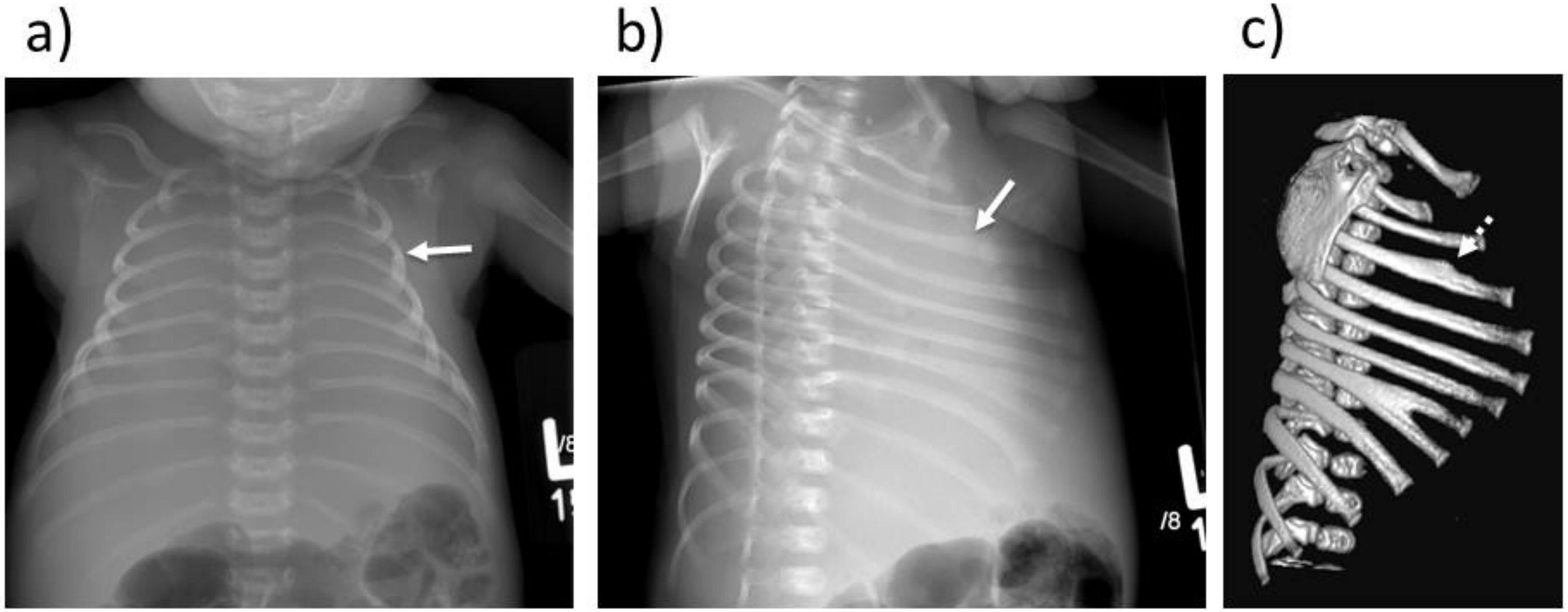

Figure S2:
Examples of false negative rib fracture diagnosis on radiography, true positive on CT (Case Code 10). Post-mortem imaging of an 11 month old male patient, obtained 2 days after death. (a) Anteroposterior radiograph (shown) and oblique views (not shown) did not reveal any rib fractures (only 3/38 (7.9%) readers reported bilateral 3rd-5th rib fractures). (b) Composite axial images from the post -mortem chest CT of different ribs (annotated), demonstrate anterior rib fractures of the right 2nd to 6th ribs and left 3rd – 5th ribs (arrows), all fractures apart from the right 6th rib fracture was confirmed at autopsy. The slight buckle of the right 6th rib therefore represents a false positive finding on CT in this case.

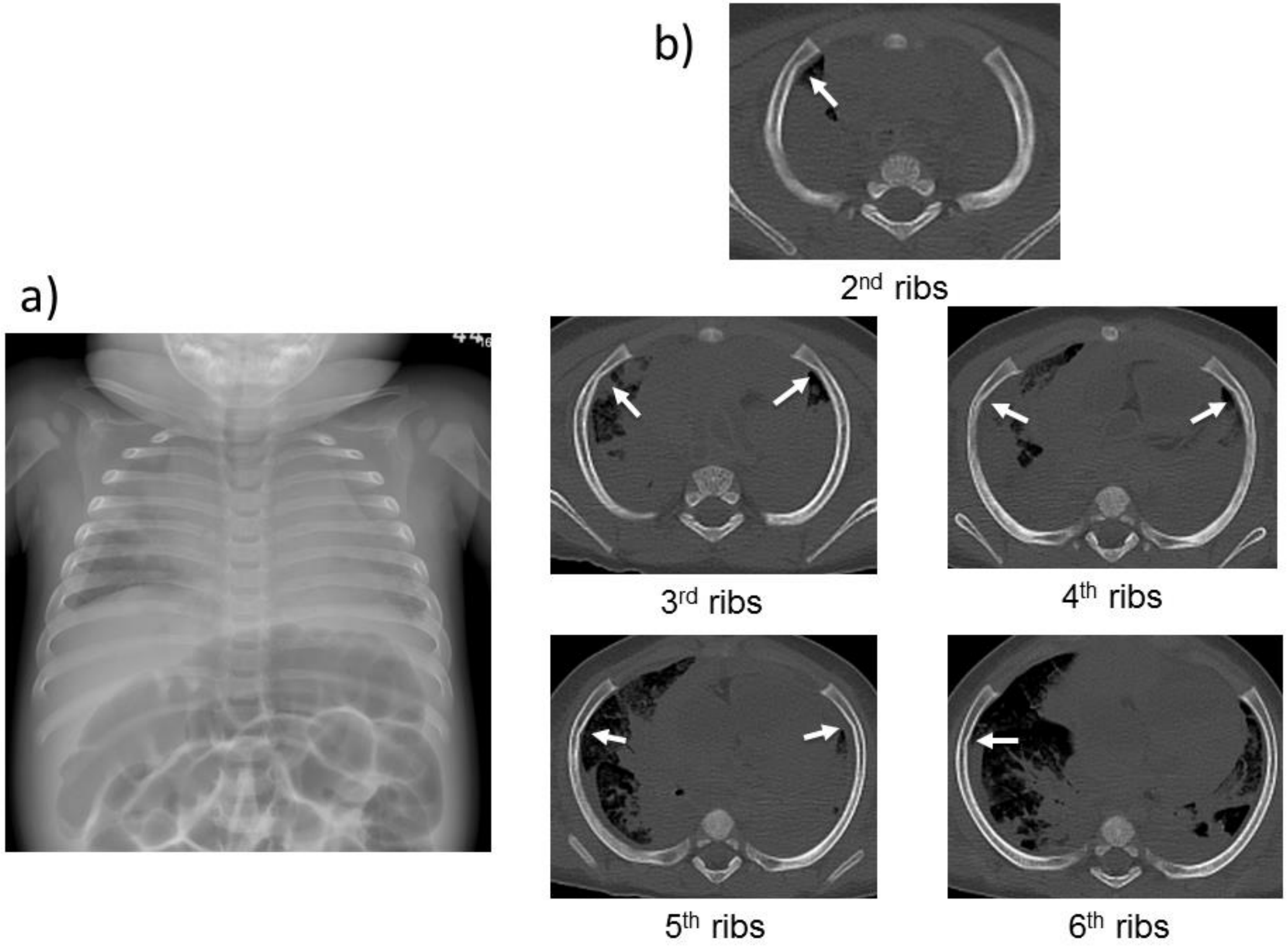

Figure S3:

Examples of false negative rib fracture diagnosis on radiography, true positive on CT (Case Code 20). Post-mortem imaging of a 1 month old female patient, obtained 5 days after death. (a) Anteroposterior radiograph (shown) and oblique views (not shown) did not reveal any rib fractures to the majority of readers (only 2/38 (5.3%) readers reported left sided anterior and posterior 2nd-7th rib fractures). (b) Composite image of multiple axial 7mm maximum intensity projection (MIP) images from the post -mortem chest CT of different ribs (annotated), demonstrate left sided anterior fractures of 2nd to 6th ribs and left posterior 1st-3rd and 5th-7th ribs (arrows). Of note, additional fractures were also reported at autopsy of the right anterior 2-4th ribs, which are difficult to see on CT and thus represent false negative findings. (c) The autopsy images of the left hemithorax, viewed from the interior aspect with left lung when extracted, shows paraspinal (white circle) and anterior thoracic haemorrhages (dashed arrows), surrounding the areas of the acute rib fractures.

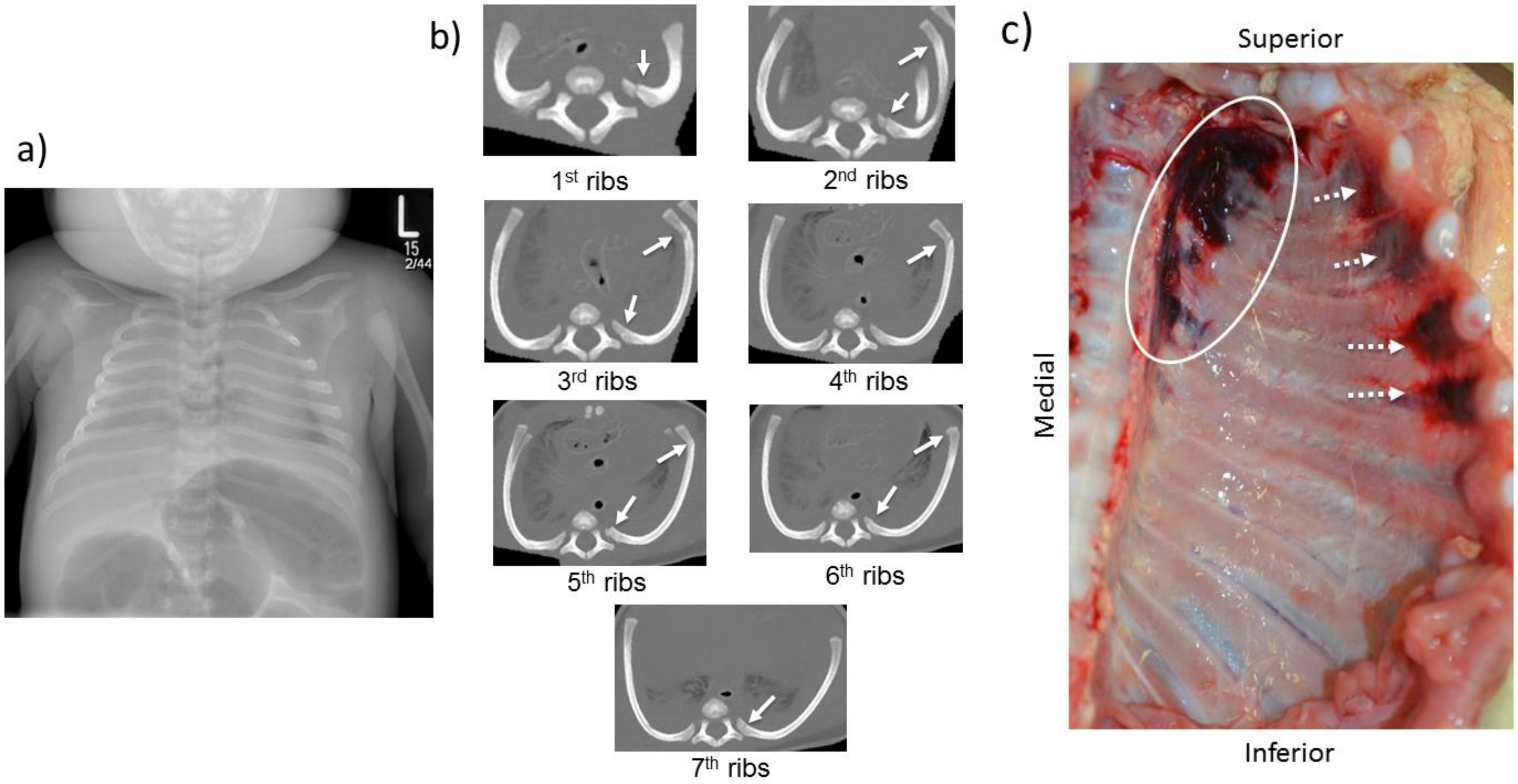

Figure S4:
Post-mortem imaging of a 4 month old female patient, obtained 4 days after death. An example of a false negative rib fracture diagnosis on CT, with true positive on radiography (Case code 24). Although the fracture can be visualised on both imaging modalities, many more participants were able to identify the fracture on the radiographic imaging. (a) Anteroposterior and (b) right oblique view radiographs revealed a right 5th rib fracture (white arrow) (seen by 24/38 readers, 63.1%). (c) Axial post-mortem CT imaging through this rib demonstrates early callus formation (white arrow) and subtle sclerosis in keeping with a healing rib fracture.

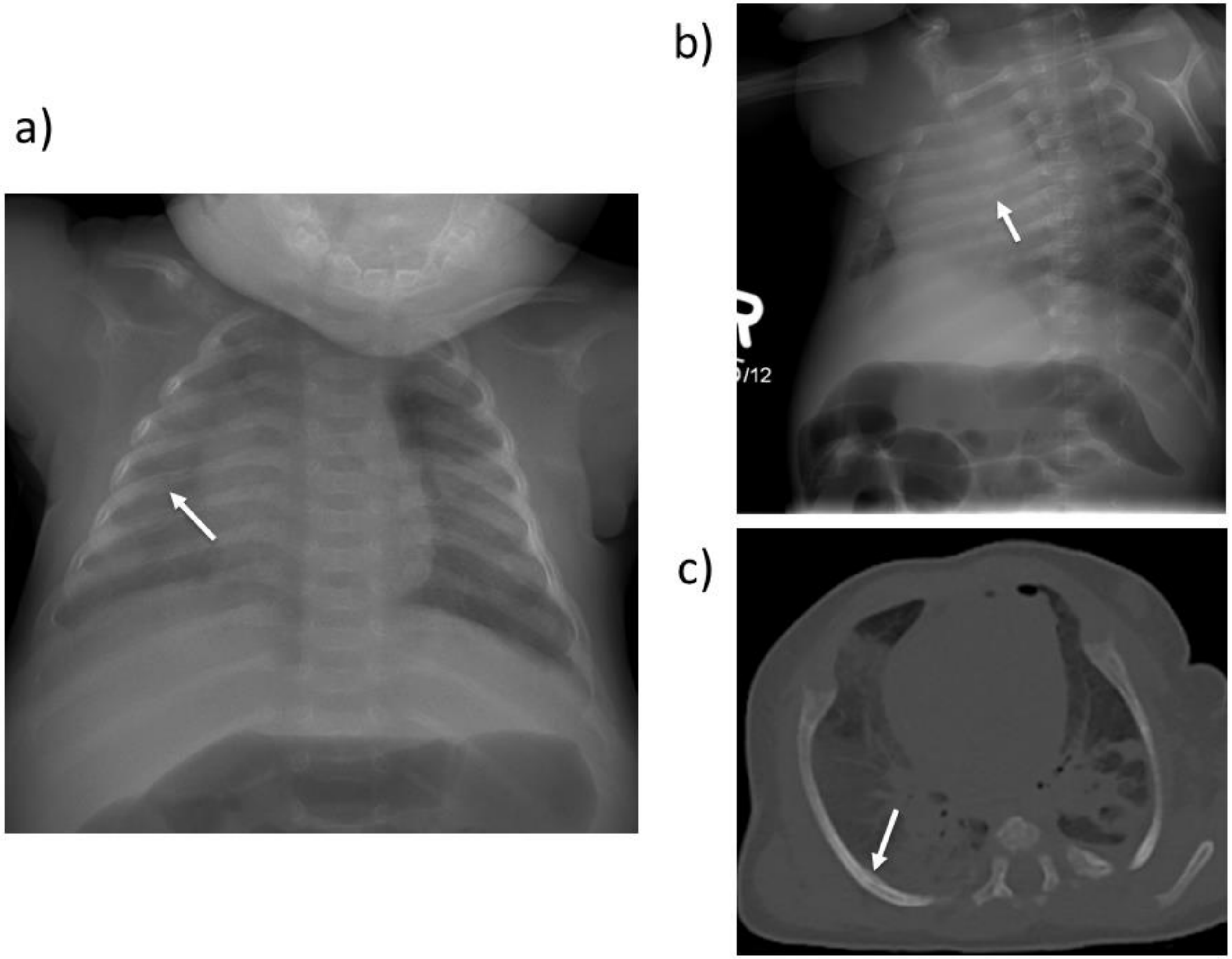

Supplement: Supplementary appendix [file mmc1.pdf]
